# Strategy to Prime the Host and Cells to Augment Therapeutic Efficacy of Progenitor Cells for Patients with Myocardial Infarction

**DOI:** 10.3389/fcvm.2016.00046

**Published:** 2016-11-24

**Authors:** Jeehoon Kang, Tae-Won Kim, Jin Hur, Hyo-Soo Kim

**Affiliations:** ^1^Department of Medicine, Seoul National University Hospital, Seoul, South Korea; ^2^Molecular Medicine & Biopharmaceutical Science, Graduate School of Convergence Science & Technology, Seoul National University, Seoul, South Korea; ^3^National Research Laboratory for Stem Cell Niche, Center for Medical Innovation, Seoul National University Hospital, Seoul, South Korea

**Keywords:** cell therapy, myocardial infarction, priming agents, peripheral blood mononuclear cells, MAGIC cell therapy

## Abstract

Cell therapy in myocardial infarction (MI) is an innovative strategy that is regarded as a rescue therapy to repair the damaged myocardium and to promote neovascularization for the ischemic border zone. Among several stem cell sources for this purpose, autologous progenitors from bone marrow or peripheral blood would be the most feasible and safest cell-source. Despite the theoretical benefit of cell therapy, this method is not widely adopted in the actual clinical practice due to its low therapeutic efficacy. Various methods have been used to augment the efficacy of cell therapy in MI, such as using different source of progenitors, genetic manipulation of cells, or priming of the cells or hosts (patients) with agents. Among these methods, the strategy to augment the therapeutic efficacy of the autologous peripheral blood mononuclear cells (PBMCs) by priming agents may be the most feasible and the safest method that can be applied directly to the clinic. In this review, we will discuss the current status and future directions of priming PBMCs or patients, as for cell therapy of MI.

## Introduction

Ischemic heart disease is one of the leading causes of death worldwide. Beyond the current practice guidelines of percutaneous coronary intervention and standard medication for acute myocardial infarction (MI; consisting of aspirin, clopidogrel, heparin, and abciximab), how to preserve or repopulate cardiomyocytes during the necrotic process of infarction has been left as an unsolved issue. Along with the boost of stem cell biology, preclinical studies have shown positive and optimistic results of stem or progenitor cells to repair ischemic limb or myocardium (1, 2), which brought great expectation that these therapies could rescue cardiomyocytes damage, enhance vascular density, and eventually rebuild the necrotic myocardium. Also, early human studies showed a decrease in the infarct size after MI by implantation of bone marrow stem cells (3). During the past decade, many clinical trials showed positive results of cell therapy (4–7), while other clinical studies showed no beneficial effect of cell therapy over placebo (8, 9). Meta-analyses have been reported, trying to give a clear answer to the question about the efficacy of stem cell therapy (10–12). These meta-analyses also have shown conflicting results due to the large heterogeneity of clinical trials of stem cell therapy (i.e., cell type, delivery mode, timing of infusion, endpoint, and follow-up period). Until now, the accumulated evidence from the relatively homogenous clinical trials, which used autologous bone marrow monocytes or peripheral blood progenitors mobilized from bone marrow for patients with acute MI, indicates that the effect of stem cell therapy is proved, but its efficacy is modest.

The theoretical background of stem cell therapy is the pluripotency and plasticity of stem cells that undergo transdifferentiation into mature cells and repair the damaged tissue (13). A wide variety of cell types are used, including bone marrow mononuclear cells (BM-MNC), endothelial progenitor cells (EPCs), peripheral blood mononuclear cells (PBMCs), peripheral blood mobilized-progenitor-cells from bone marrow (PB-MPCs), mesenchymal stem cells, cardiac stem cells, etc. Also, various methods are used to augment the efficacy of stem cell therapy, such as genetic manipulation, or non-genetic cytokine/chemokine priming the cells/hosts, or *ex vivo* expansion of cells. Genetic manipulation can be performed either by direct transfer of genes into the host (using retroviruses or adenoviruses) or by using living cells as vehicles to transport the genes of interest. Priming can be done with various cytokines/chemokines, by direct injection to the host or by *ex vivo* application of the priming agent on cells. After preparation of stem cells, these cells can be delivered directly to the damaged tissue, by systemic injection or by intracoronary injection in the case of ischemic heart disease (14).

Among various methods for stem cell therapy, PB-MPCs are the most feasible and practical cell type, due to the comparable efficacy to bone marrow progenitors and the non-invasive method of collection compared to bone marrow progenitors. However, PB-MPCs have shown limited efficacy, probably owing to the low homing-efficiency, the poor long-term survival rate of infused cells, and the potential dysfunction of PB-MPCs (15, 16). In this review, we will discuss a method to enhance the therapeutic efficacy of PB-MPCs, called “priming,” and the various non-genetic agents/conditions used to prime the infused cells or the patients themselves. Also, we will introduce recent clinical trials and ongoing trials for stem cell therapy in MI, along with a current trial conducted by our institute.

## Rationale for Cell Therapy in MI

After an ischemic insult in the myocardium, endogenous repair would be minimal or insufficient. The various cell types including cardiomyocytes and stem cells within or out of heart participate in this endogenous repair process (17). However, this is not sufficient to prevent deleterious remodeling, leading researchers to pursue exogenous cell delivery to achieve the substantial degree of cardiac regeneration. The best-case scenario would be that the delivered cells differentiate into functional cardiomyocytes and replace the necrotic tissue, which turned out to be unachievable due to the low retention rate and limited differential potential of injected cells (18). Therefore, the aim of current cell therapy has been established to improve myocardial perfusion through neovascularization, modulate the inflammatory response by ischemia, and correct metabolic and electromechanical disturbances (19). Currently, it is well recognized that the prominent mechanism of the beneficial effect of cell therapy involves the activation of endogenous healing pathways through paracrine factors. These pathways can improve the survival of cardiomyocytes and activate recruitment of endogenous stem cells (17). Also, cell therapy aids angiogenesis to the damaged myocardium by either direct differentiation or by activating endogenous angiogenic progenitors (20). Overall, the goal for cell therapy is more to achieve a niche favorable for regeneration, rather than for direct differentiation to cardiomyocytes.

## Various Priming Agents for PBMCs or PB-MPCs

**Figure 1:**
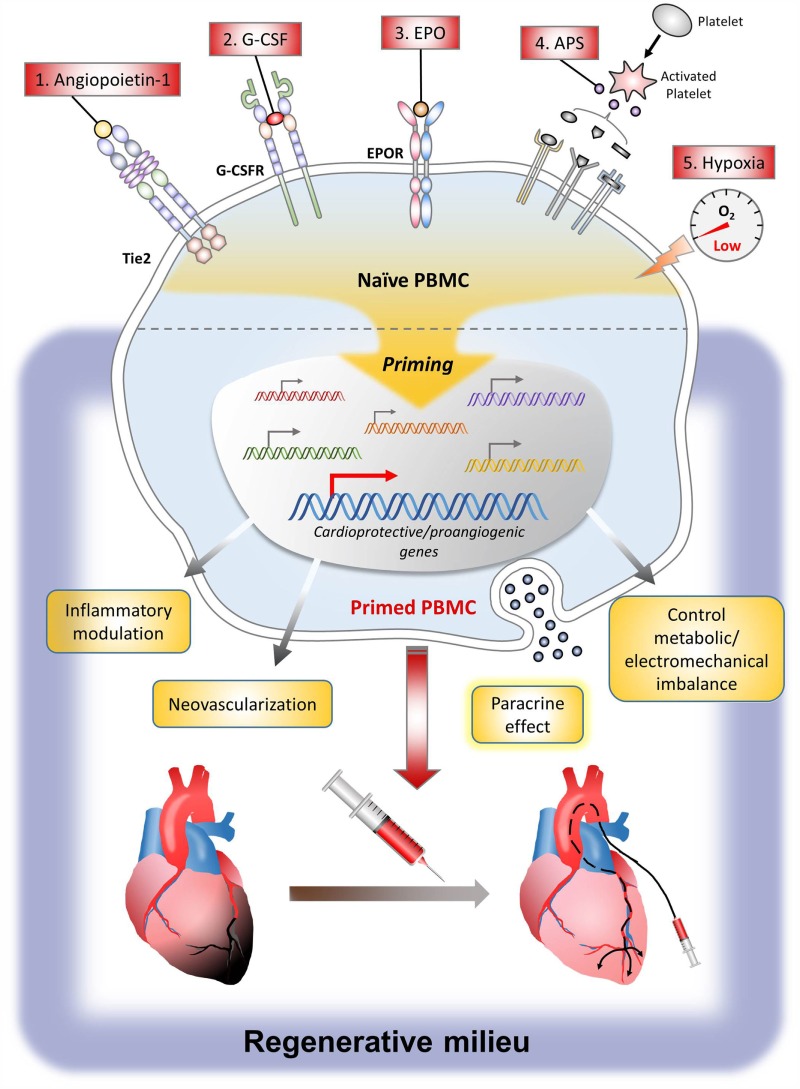
**Scheme of the cellular effects of priming agents**.

Currently, various cell types have been studied for cell therapy in MI. Among them, the most commonly used cells are BM-MNCs, PBMCs, or PB-MPCs containing stem cells mobilized from bone marrow by mobilizers such as subcutaneous injection of granulocyte colony-stimulating factor (G-CSF) (21). To augment the therapeutic efficacy of these cells, strategy to prime the cells by direct exposure to the priming agent or to prime the patients by systemic administration of the priming agent is a useful and practical method in the clinic. Major priming agents include G-CSF, angiopoietin-1 (Ang-1), erythropoietin (EPO), activated platelet supernatant (APS), growth factors such as SDF-1 and vascular endothelial growth factor (VEGF), and conditions such as hypoxia. The action mechanism of these agents is the induction of many genes that can induce angiogenesis, control inflammation, and promote tissue regeneration, leading to the enhanced therapeutic efficacy of stem cells. The following are preclinical study results explaining the mechanism of major agents or conditions that have been used for priming of cell or host (Figure 1).

### Granulocyte Colony-Stimulating Factor

Granulocyte colony-stimulating factor is a well-known agent that potently mobilizes hematopoietic stem cells from the bone marrow (22). G-CSF acts *via* the activation of the G-CSF receptor, which initiates maturation, survival, proliferation, and functional activation of granulocytes (23). The most critical factor determining the therapeutic efficacy of mobilization using G-CSF may be the homing of mobilized PBMCs or PB-MPCs, which is mediated by the SDF-1/CXCR4 axis (24). Several mechanisms have been reported for G-CSF itself to help repair the damaged myocardium, such as, apoptosis inhibition (25), induction of angiogenesis (26), anti-inflammatory effects (27), modulation of extracellular matrix (28), and many other paracrine effects (29).

Until now, several human clinical trials have evaluated the safety and efficacy of G-CSF injection in patients with acute MI, where they tested the effect of G-CSF to prime the host or patient, but not the effect of cell-priming or mobilization effect. Some trials have shown positive results (30–32), while others have failed to confirm the above beneficial effects of G-CSF in patients with acute MI (33–35). Disappointingly, meta-analyses showed that G-CSF therapy was not associated with any significant benefit in left ventricular systolic function, whereas subgroup analyses suggested that G-CSF therapy might be beneficial in selected patients (36, 37). But, these negative or conflicting results do not deny the efficacy of G-CSF as a mobilizer of progenitor from bone marrow to peripheral blood or as a cell-priming agent.

### Angiopoietin-1

Angiopoietin-1 is a growth factor binding to the Tie2 receptor expressed on endothelial cells and hematopoietic stem cells. Through the Tie2 signaling, Ang-1 plays an essential role in postnatal angiogenesis by mediating vessel maturation and maintaining vessel integrity (38). In a previous preclinical study, we found that most of the PB-MPCs had Tie2 receptor and that short-term Ang-1 exposure induced PB-MPCs to differentiate into endothelial lineage through the Tie2/Ets-1 signaling pathway. This eventually enhanced the neovasculogenic potential in the ischemic tissue (39). Additionally, short-term Ang-1 priming induced PB-MPCs to express α4, α5, and β1 integrins that were also downstream targets of Ets-1. These *in vitro* results are important because the weakest point of intra-arterial delivery of progenitors to myocardium is the poor retention rate. PB-MPCs that were primed with Ang-1 become “sticky,” leading to significant improvement of retention efficiency and therapeutic efficacy after intra-arterial delivery. The process of applying Ang-1 to the clinic is ongoing by producing human-grade Ang-1 protein in an economically feasible way.

### Erythropoietin

Erythropoietin, a glycoprotein hormone produced primarily by the kidney, is a well-known cytokine that controls erythropoiesis (40). Therapeutically, EPO is commonly used as a treatment for anemia in patents with renal failure or hematologic disorders. At the cellular level, EPO acts through the EPO receptor, which changes its conformation upon binding with EPO. This results in phosphorylation of JAK2 kinases, leading to the activation of numerous intracellular signaling cascades, such as the JAK/STAT, PI3-kinase/Akt, and MAPK pathway (41).

Due to its cellular effect (i.e., enhanced proliferative, vasculogenic, and anti-apoptotic properties) (42), EPO has been used in clinical trials as a host-priming agent in MI patients, so as to decrease infarct size and preserve cardiac function. However, the results were disappointing, showing conclusions that EPO did not reduce myocardial infarct size (43–45). This discrepancy between the positive cellular effect and negative results in clinical trials can be explained by several hypotheses; the insufficient local concentration at the infarcted myocardium and the unwanted systemic effects of EPO (46, 47).

Based on these facts, our group performed a preclinical study using EPO as a cell-priming agent for PBMCs or PB-MPCs (48). With adoption of an *ex vivo* cell-priming strategy, we expected several benefits; to maximize the cellular effect of EPO on target cells, while avoiding the systemic side effects of EPO. As a result, cell-priming with EPO induced a shift in monocytes polarization toward CD14(++)/CD16(+) monocytes, which are so-called vasculogenic/anti-inflammatory monocytes that play a pivotal pro-healing action in debris scavenging, wound healing, and angiogenesis (49). Also, EPO-primed PBMCs could upregulate expression of integrins, which could enhance homing to the infarcted myocardium. In a paracrine matter, EPO-primed PBMCs secreted cytokines such as IL8 and IL10, to form a vasculogenic niche at the target ischemic tissue. Taken together, our results showed that *ex vivo* EPO-priming augmented the vasculogenic potential of human PBMCs, proving to be a promising and practical method to augment the therapeutic efficacy of PBMCs in cell therapy.

### Activated Platelet Supernatant

Platelets, which are known to play a role in hemostasis, simultaneously promote tissue repair *via* releasing a vast amount of cytokines and chemokines that favor angiogenesis and wound healing (50). In previous studies, autologous platelets have been used in human clinical trials for bone repair (51), wound healing in ocular surface disease (52), and for cardioprotection from ischemia–reperfusion injury (53). Immediately after an ischemic injury, platelets play a key role in the surge of local cytokines and chemokines, which recruit monocytes to the damaged tissue, stimulate endothelial cell proliferation, and increase vascular permeability (54).

Based on this background, we used the surge of cytokines and chemokines from platelets, the so-called APS, as a priming agent for human PBMCs or PB-MPCs in a previous preclinical study. We could find that APS-primed PBMCs were polarized to M2 monocytes, which could efficiently induce gene expression of angiogenic molecules. Furthermore, APS priming could promote angiogenesis in a paracrine manner, by secreting angiogenic cytokines, such as IL8, IL10, and PDGF (55). Also, in a rat MI model, APS-primed PBMCs could decrease fibrosis area and myocardium wall thinning, which leads to improvement in cardiac function (56).

### Hypoxia

Although not exactly an “agent” but rather a “condition,” hypoxia has been used to augment the efficacy of cell therapy. In various preclinical studies, hypoxia could direct embryonic stem cells or PBMCs to differentiate into cardiomyocytes (57), chondrocytes (58), or vascular progenitor cells. In previous preclinical studies, hypoxia priming enhanced the differentiation of embryoid bodies into meso-endodermal cells, which differentiated into vascular-lineage cells more efficiently than normoxic embryoid bodies (59). Also, hypoxic preconditioning to cardiosphere-derived cell monolayer sheets, increasing the expression of VEGF through the PI3-kinase/Akt signaling pathway, which leads to improved left ventricular function in chronically infarcted hearts (60). Although ischemic preconditioning has been proven to be beneficial, applying this to the clinic is difficult due to the inability to predict the onset of ischemia in the apparently normal persons.

### Other Growth Factors

Other than the priming agents/conditions listed above, various agents have been studied to enhance the therapeutic efficacy of stem cell therapy in MI.

### Vascular Endothelial Growth Factor

Initial research of VEGF, which plays a critical role in angiogenesis (61), was promising in preclinical studies as a therapeutic agent for ischemic disorders. However, clinical trials failed, only to reveal that VEGF offered no improvement in treated patients as compared with placebo (62). These results may have been partially attributed to the short-lived effect and high instability of the protein when injected as a bolus (63). Currently, different methods to deliver VEGF, such as using scaffolds or other biomaterials, are under research.

### SDF-1

The interaction between SDF-1 and CXCR4 plays an important role in vasculogenesis especially for the engraftment and maintenance *in situ* (64). In preclinical studies, SDF-1 priming of EPCs could enhance adhesion and extravasation of progenitor cells to ischemic sites, promoting firm adherence to activated endothelium (65) and effective to enhance cardioprotective effect in animal experiments (66), which need to be confirmed in human studies.

### Quality and Quantity Culture

Due to the limited effect of single agents, there have been trials that used multiple agents for priming. In a previous animal study, EPC populations, such as CD34+ and CD133+ cells, could be enriched by the method for quality and quantity-control culture (QQ culture; a combination of stem cell factor, thrombopoietin, Flt-3 ligand, VEGF, and interleukin-6). Also, priming monocytes under QQ culture induced anti-inflammatory and angiogenic monocytes/helper T lymphocytes (67).

## Recent Clinical Trials for Stem Cell Therapy in MI

Along with a vast amount of preclinical studies, various human clinical trials have tested the efficacy of stem cell therapy in MI. The results varied even though they were conducted under contemporary treatment strategies for MI during the past decade (6, 8, 9, 68–72). Some studies reported positive effects of stem cell therapy, while others failed (Table 1). These conflicting results in clinical studies in contrast to the positive results of preclinical ones emphasize the necessity to augment therapeutic efficacy of stem cell therapy by refining the protocol.

**Table 1:** **Recent human clinical trials for stem cell therapy in MI**.

Name of study, reference	Cell type	Patients enrolled	Follow-up (months)	Results
FINCELL, Huikuri et al. (68)	BM-MNC	STEMI patients	6	Improvement in LVEF
				No difference in adverse clinical events
REGENT, Tendera et al. (69)	Unselected BM-MNC and selected [CD34(+) CXCR4(+)] BM-MNC	Acute MI with LVEF <40%	6	No difference in changes of LVEF, left ventricular end-systolic volume, and left ventricular end-diastolic volume (significant increase of LVEF subgroup of patients with severe LVEF impairment)
				No difference in major cardiovascular event (death, reinfarction, stroke, target vessel revascularization)
BONAMI, Roncalli et al. (70)	Autologous BM cells	Acute MI patients	3	Improvement in myocardial viability
LateTIME, Traverse et al. (9)	BM-MNC	MI patients	6	No difference in LVEF, wall motion abnormality of the infarct zone, and border zone
				No significant change in LV volumes and infarct volumes
APOLLO, Houtgraaf et al. (72)	Adipose tissue-derived cells	STEMI patients	6	Positive trend toward improved cardiac function, perfusion defect
				50% reduction of myocardial scar formation
				No severe adverse events
CADUCEUS, Makkar et al. (6)	Cardiosphere-derived cells	MI patients	6	Reduction in scar mass
				Increase in viable heart mass, regional contractility, and regional systolic wall thickening by MRI imaging
				No change in end-diastolic volume, end-systolic volume, and LVEF
TIME, Traverse et al. (71)	BM-MNC	STEMI patients with LV dysfunction	6	No difference in increase of LVEF or global left ventricular function
SWISS-AMI, Sürder D et al. (8)	BM-MNC	STEMI patients	4	No improvement in LV function

*BM-MNC, bone marrow mononuclear cells; LV, left ventricular; LVEF, left ventricular ejection fraction; STEMI, ST segment elevation myocardial infarction*.

There are several ongoing clinical studies of cell therapy for acute MI, which may report results within the next couple of years (searched from www.clinicaltrials.gov, using keywords of “stem cell” and “myocardial infarction”). These studies include the EXpanded CELL ENdocardiac Transplantation (EXCELLENT) trial (NCT02669810), which will evaluate the efficacy of intracardiac injection of ProtheraCytes (autologous PB-CD34+ stem cells after automated *ex vivo* expansion with the StemXpand machine), the A Randomized, Open labEled, muLticenter Trial for Safety and Efficacy of Intracoronary Adult Human Mesenchymal stEm Cells Acute Myocardial inFarction (RELIEF) study (NCT01652209), which will evaluate the efficacy of adult human mesenchymal stem cells, and the Enhanced Angiogenic Cell Therapy – Acute Myocardial Infarction (ENACT-AMI) trial (NCT00936819), which uses autologous progenitor cells by overexpressing eNOS to enhance the function of autologous progenitor cells. Also, the BAMI (The Effect of Intracoronary Reinfusion of Bone Marrow-derived Mononuclear Cells on All-Cause Mortality in Acute Myocardial Infarction) trial (NCT01569178) is currently ongoing to demonstrate whether a single intracoronary infusion of autologous BM-MNC is safe and reduces all-cause mortality in patients with reduced left ventricular ejection fraction after successful reperfusion for acute MI. There trials may give us more specific answers for the efficacy of stem cell treatment on AMI.

## The Unique Combi-Cytokine-Based Autologous PB-MPCs Therapy for Patients with Acute MI: Magic Cell Therapy

During the past 15 years, our institute has performed a series of clinical trials of cytokine-based cell therapy, named the MAGIC cell (Myocardial Regeneration and Angiogenesis in Myocardial Infarction with G-CSF and Intracoronary Stem Cell Infusion) trial. By a series of work, we could demonstrate that intracoronary infusion of PB-MPCs in MI patients is safe and effective in improving cardiac function (7) with persistent efficacy in a 5-year follow-up (73). The plausible mechanism of long-term efficacy comes from the pro-healing effect of cell therapy on the coronary arterial segment implanted with drug-eluting stent (74). Currently, the MAGIC cell therapy has been approved by the Korean government and is being performed in tertiary hospitals. The PB-MPCs that we are using in this MAGIC cell protocol are primed by multiple agents/conditions, based on the results from previous study results.

First, the patient and PB-MPCs are primed *in vivo* by EPO and G-CSF; each are injected to the patient by (1) intravenous infusion of 4.5 μg/kg darbepoetin (long-acting EPO), (2) subcutaneous G-CSF at 5 μg/kg body weight twice daily for 3 days. Second, priming with autologous APS and hypoxia is achieved in the apheresis process. PB-MPCs are collected under an apheresis system [minimum target cell dose is 2 × 10^9^ monocytes and 7 × 10^6^ CD34(+) cells/patient] and by using the mononuclear cell collection method, not only PB-MPCs but also platelets are collected. Platelets are activated by the apheresis process, forming an autologous APS. Therefore, during the short incubation period within the sealed apheresis package, PB-MPCs are primed by APS and also by hypoxia. Overall, we combined various priming/conditions so as to maximize the therapeutic efficacy of PB-MPC cell therapy. The beneficial cellular effects of each single priming agent, based on preclinical studies, are shown in Table 2.

**Table 2:** **Study results of priming agents for peripheral blood mononuclear cells or peripheral blood mobilized-progenitor cells from the bone marrow**.

Priming agent and priming method	Cell species	Animal model	Outcome	Reference
Agent> angiopoietin-1 (Ang-1) Method> primed with COMP-Ang-1 (400 ng/ml) for 2–4 h	PBMCs/PB-MPCs from acute myocardial infarction patients	Rabbit ear ischemia and reperfusion model Athymic nude mouse hind limb ischemia model	Increased expression of endothelial cell markers (CD31 and VE-cadherin) and adhesion molecules (integrin α4, α5, and β1) Increased Matrigel tube formation and incorporation ability Enhanced first-pass engraftment into the distal vascular bed and enhances neovascularization of the ischemic area (*Animal model*)	(34)
Agent> erythropoietin Method> primed with Human recombinant EPO (10 IU/ml) for 6 h	PBMCs/PB-MPCs from healthy volunteers after 3-day subcutaneous injection of G-CSF (10 μg/kg)	Athymic nude mouse hind limb ischemia model and myocardial infarction model	Increased synthesis of vasculogenesis-related cytokines and integrins (IL8, IL10, bFGF, PDGF, MMP2, integrin αV, β1, β2, and β8) Increased proliferation of CD14(++)/CD16(+) angiogenic mononuclear cells and reduced apoptotic cells Enhance neovascularization in ischemic limb and repair myocardium after infarction through cellular and humoral mechanisms (*Animal model*)	(43)
Agent> G-CSF Method> *in vivo* injection	Rabbit	Rabbit myocardial infarction model	Upregulation of VEGF, MMP-1, SDF-1 expression within infarcted area (*Animal model*) Increased CXCR4(+) bone marrow cells and macrophages to infarcted area (*Animal model*) Reduction of scar area in myocardial infarction model	(19)
Agent> activated platelet supernatant (APS) Method> primed with APS for 6 h	PBMCs/PB-MPCs from healthy volunteers after a 3-day subcutaneous injection of G-CSF (10 μg/kg)	Athymic nude mouse hind limb ischemia model	Increased gene expression of cytokines (i.e., IL8, IL10, IL13, IL17, bFGF, and TNFα) Increased CD34(+), CD31(+), Tie2(+), CXCR4(+) cells Increased proliferation of CD14(++)/CD16(+) angiogenic mononuclear cells and reduced apoptotic cells Enhanced adhesion and migration activity Increased tissue regeneration and angiogenesis (*Animal model*)	(50)

*COMP, cartilage oligomeric matrix protein; PBMC, peripheral blood mononuclear cell; PB-MPCs, peripheral blood mobilized-progenitor-cells from bone marrow*.

## Strength and Limitations of Cell Therapy in Ischemic Heart Disease

Stem cells are ideal candidates for use in regenerative medicine because of their ability to differentiate to multiple cell lineages. In the case of ischemic heart diseases, stem cell therapy could regenerate the damaged myocardial or vascular tissue and/or prevent adverse ventricular remodeling after infarction. Current options for reperfusion (e.g., medical treatment, percutaneous coronary intervention, and surgical treatment) have significantly improved outcomes after MI; however, these techniques do not reverse the necrosis process after ischemia. In this aspect, stem cell therapy may provide a unique additional treatment for MI.

Despite the advantages of stem cell therapy, it is important to point out the limitations, so as to prevent unnecessary optimism. First, stem cells are not fully under control; adult stem cells are difficult to expand in culture, whereas embryonic stem cells have the risk of chromosomal abnormalities (75). Sufficient expansion is needed for treatment efficacy, whereas chromosomal abnormalities have the risk of teratoma formation. Also, the adequate type/timing and number of cells delivered at the site of engraftment has not been fully evaluated. *Ex vivo* expansion of injected cells may be a method to increase efficacy; however, this is a procedure prone to contamination. The most fundamental limitation may be that the mechanism of stem cell therapy is not yet fully understood. Unlike the initial expectation that injected stem cells may engraft and transdifferentiate into myocardial cells based on the pluripotency and plasticity, it is understood that the positive effects on cardiac function may have resulted from a paracrine effect (48).

## Conclusion and Future Directions

Despite the promising results of cell therapy in preclinical studies, human trials for stem cell therapy in MI patients have shown marginal benefits compared to placebo. However, by understanding the precise mechanism of cardiac repair and by understanding the limitations of current methods for cell therapy, we are observing slow and steady progress. Among the various strategies currently available, priming cells or the host is the most feasible and practical method that can be used at the clinic. Single agents have shown marginal effects, and combination of multiple priming agents may have beneficial effects. Furthermore, future studies are needed to better define the crucial mechanism of cardiac repair after cell therapy. Identification of the optimal cell type and clarification of the optimal manipulation to the cell is essential for advance in cell therapy for MI.

## Author Contributions

All the authors researched data for the article, discussed its content, and wrote, edited, and reviewed the manuscript.

## Conflict of Interest Statement

All authors declare no conflicts of interest, financial or otherwise, in the writing of this manuscript.
